# Wilhelm Waldeyer—An Important Scientific Researcher of the 19th Century in the Context of His Memoirs and Major Monographies

**DOI:** 10.3389/fsurg.2021.752709

**Published:** 2021-11-01

**Authors:** Hubert Scheuerlein, Carolina Pape-Köhler, Ferdinand Köckerling

**Affiliations:** ^1^Academic Teaching Hospital of the University of Göttingen, Clinic for General, Visceral and Paediatric Surgery, St. Vincenz Hospital Paderborn, Paderborn, Germany; ^2^Department of Surgery and Center for Minimally Invasive Surgery, Academic Teaching Hospital of Charité Medical School, Vivantes Hospital, Berlin, Germany

**Keywords:** Waldeyer, 100th death anniversary, autobiography, Ovary and Egg, The Pelvis

## Abstract

Wilhelm Waldeyer was one of the most important anatomists of his time. The year 2021 marks the 100th anniversary of his death. His name not only lives on in terms such as “Waldeyer's pharyngeal ring” or “Waldeyer's fascia,” he also coined the terms “neuron” and “chromosome.” He produced monumental monographies such as “The Pelvis” and “Ovary and Egg”. Waldeyer's legacy is a large body of lifetime work that continues to impress to this day. However, he also published works that today would be described as racist. His view of a woman's role was and is also controversial. Nevertheless, reading his autobiography (*Lebenserinnerungen*) today is still beneficial because it vividly illustrates the academic life and a scholarly existence of that era.

## A Brief Overview of His Curriculum Vitae and Oeuvre

Heinrich Wilhelm Gottfried Waldeyer, known from 1916 as Wilhelm von Waldeyer-Hartz, was born in Hehlen an der Weser (Kreis Holzminden) in 1836. He spent his youth in the Paderborn region and repeatedly made a point of disclosing with pride his descent from a Westphalian farming family (1). In October 1856, he took up a place at the University of Göttingen with the aim of studying mathematics and natural sciences. After two semesters, he made the acquaintance of the anatomist Jakob Henle. He was so fascinated by Henle that in 1857 he transferred to study medicine. Already at this early time, he had made the decision to become a university lecturer in anatomy. “He continued his studies in Greifswald and Berlin, where he graduated in 1862” (2, 3). “He started his career in physiology in Königsberg and Breslau”. In 1867 he was appointed “full professor of pathological anatomy in Breslau”, “still waiting for an opportunity to enter anatomy, his desired specialty” (2). “The Franco-German War in 1870/1871 saw Waldeyer working as a military surgeon.” In 1872 he was appointed to full professorship of anatomy in Strasbourg. This period in Strasbourg was the best time of his life (1, 4). He declined calls to Vienna, Bonn and Munich (1). “In 1883, he moved to the prestigious Chair of Anatomy in Berlin” (2). He remained there for the unusually long period of 33.5 years, as full Professor of Anatomy and Director of the Anatomical Institute. His great talent as a teacher ensured that his lectures were always filled to the brim (1). He “was married and had four children” (2). His honorary titles, honorary memberships of scientific societies and academies, nominations, honours and accolades are very numerous. During his long tenure in Berlin, he was deacon and rector magnificus several times. In 1912, he was called to the (Prussian) House of Lords. In 1916, on the occasion of his resignation, he received a hereditary peerage: he kept the memory of his mother, née von Hartz, in his ennoblement (“von Waldeyer-Hartz”). Waldeyer remained at the University of Berlin until the age of 80, with all the duties that this position imposed (5). According to Sobotta, he passed away peacefully after a cerebral apoplexy on 23rd January 1921.

“One can only marvel as to how it was possible for a man such as Waldeyer, who was already snowed under with work, to develop such multilateral tasks. He belonged to those lucky characters who do not complain about the amount of work they perform but feel even more fulfilled, the more work they perform” (1). “Waldeyer published 269 papers throughout his career. These span all regions of the human body and an impressive range of themes: gross anatomy, histology, physiology, pathology, anthropology, education, history, arts, and macroscopic and histologic technique” (2).

Among his works are monumental monographies such as “The Pelvis” and “Ovary and Egg,” which is probably his most important work; both of these have been reprinted up to the present time. Numerous anatomical terms were or still are associated with his name.

“He was obviously a generalist, and his scientific merits lie not so much in his original research, but in his ability to summarise and give a name to new scientific concepts” (2). In this context Waldeyer today is remembered as one of the founders of the neuron theory, coining the term “neuron” to describe the cellular function unit of the nervous system and enunciating and clarifying that concept in 1891. He also coined the term “chromosome” (1888) to describe the bodies in the nucleus of cells (6).

Waldeyer's publications include articles about human specimens of African origin (2). The publication years “correspond to the height of German colonialism between 1884 and 1914”. He “examined African brains above all, but also skulls and genital organs”. “Reading his descriptions today, it is difficult to accept that in this research” he “would not go beyond the racial stereotypes of his time” (2).

## Neuron and Neuron Theory

Waldeyer coined the term “neuron” and is classed as one the founders of the so-called neuron theory or neuron doctrine (5, 7, 8). The Greek word “neuron” means “tendon, sinew, ligament; nerve” and is anciently related to and means the same as the Latin word “nervous.” According to today's perception, Waldeyer's role in the acceptance of the neuron theory was not straightforward, as there was a list of research sources which preferred other terminology (9). The first description is attributed to the Swedish scientist and philosophist Emmanuel Swedenborg: Neuron—a nerve cell with its extensions. Ehrenberg, Remak, Purkinje, Deiters, Schultze, Golgi, His, Forel, Freud, Nansen, Cajal and others delivered significant research results with reference to the nerve cell and the nervous system (9, 10).

Waldeyer's contributions opposed the then-prevailing view that the brain was an interconnected network of nerves (11) and heralded, as it were, a surge in the development of neuroscience.

In 1873, Camillo Golgi (1843-1926) devised the silver nitrate method of staining nerve tissue. The nitrate stain proved the existence of a specific kind of nerve cell (later known as Golgi cells). Based on this, Waldeyer postulated that the nerve cell is the basic structural unit of the nervous system. This concept was then established and elaborated by Ramon y Cajal (5). Golgi and Cajal were Nobel Laureates in 1906 “in recognition of their work on the structure of the nervous system.” However, the two were adversaries. While Cajal assumed that the brain was made up of individual autonomous nerve cells called neurons, Golgi believed that the nerve fibres formed a continuous network similar to the blood circulation (reticulum theory). The feud between the two was so bitter that Golgi could not refrain from picking apart Cajal's theory in his Nobel Prize speech (11). Later, in his autobiography, Cajal judged Golgi to have been one of the most conceited and self-adulating gifted men he had ever known (11).

Golgi (and other main representatives like Nissl, Apáthy, Held, and Bethe) conceived the association of pathways as a product of a “syncytium,” while the representatives of the neuron theory—besides Cajal and Waldeyer, these were, e.g., His, Kölliker and Retzius—claimed the functional independence of neurons. They thus pursued the principles of reductionism (a system is completely determined by its individual parts), whereas Golgi pursued the principles of wholeness (12). The opinion of Cajal and Waldeyer has become accepted as anatomically justifiable and largely accurate. Thus, in his last classic formulation published in 1935, Cajal was able to establish six main principles as the basis of neuron theory: each neuron is an anatomical, genetic, functional, trophic, nosological, and electrophysiological unit (12). However, due to, e.g., developmental relationships between neuronal centres and periphery, concerns about a strict interpretation of the neuron theory have since emerged. Waldeyer's claim that each neuron is also a genetic unit (corresponding to Cajal's Main Theorem No. 2) also failed to gain acceptance in this way (12).

Even if the view that the neuron is the smallest specific unit of the nervous system, the building block, is generally shared today, this should not lead to an absolutisation of opposing viewpoints and perspectives (reductionist vs. holistic atomist) or to the application of a mechanical paradigm (12). Rather, from a functional point of view, the interaction of neuronal cell assemblies plays an essential role in the accomplishment of specialised tasks. Different types of networks serve different requirements and the individual neuron adapts to the demands of such networked tasks by adjusting or changing the synaptic weight according to the conditions (incoming signals are passed on or not passed on). The interaction of neurons not only gains importance with respect to the definition of neuronal centres and the understanding of their structure, but is also of particular importance for the comprehension of functional relationships in neuropsychological syndromes. In this respect, the opposing viewpoints of association psychology (reductionism) and *Gestalt* psychology (in the holistic sense) can be seen as objects of productive debate and enhancement. The self-organising mode of operation of neuronal networks cannot solely be grasped by the ideas of a machine paradigm (12–14).

Both the cell theory (Schwann 1839, Virchow 1855) and also the neuron theory (Waldeyer 1891, Cajal 1906) are, from a historical perspective, the result of technical and conceptual advancement. Both had to gain acceptance in competition with the dogmata which had been valid until that point. Until the middle of the last century, the neuron theory based on the cell theory focused on the interneural communication in the field of tension of the Golgi continuity and the concept of contiguity. In contrast to the cell theory, which is still of the utmost importance in every field of biology, the meaning of the neuron theory fades somewhat when confronted with the current developments in neurosciences (15). At the beginning of the 20th century, however, the cornerstones of the neuron theory remained stable and credible in view of the new conceptual contributions, for example, from Herrick or Heidenhain (9).

At the end of the 19th century, Waldeyer's inventive creativity on introduction of the neuron theory expresses itself in the collection, analysis and further conceptual processing of the available scientific results.

## Autobiography

In his *Lebenserinnerungen* (memoirs) Waldeyer leaves us a colourful picture of his fulfilled life as a scientific researcher and scholar (4). It contains many motto-like phrases that still have meaning today, for example: “The worst thing we could do would be to complain, accuse and despair; the right thing is to endure, learn and maintain courage!” (Preface, p. V). Waldeyer knows how to write in a very pleasing and entertaining way; he has a flair for the anecdotal and the dramatic. The language is expressive and clear; it feels immediate and is not affected by mannerisms. The more than 400 pages are preceded by a clear and plausible structure including subheadings, so that even the reader who does not wish to digest the entire work can pick out the individual pieces of the mosaic that interest him. Of course, the work must be read and understood in the context of its time. Some things seem incomprehensible or exaggerated today, such as certain nationalistic formulations, his view of the question of women and coeducation, which from today's perspective is antiquated, or his advocacy of the death penalty (p. 87).

On the first ~100 pages, he describes his family origins, his childhood and adolescence, his high school and student years (Paderborn, Göttingen, Greifswald, Berlin). Here—at the end of his life—he recalls his childhood and youth, and the elegance and vividness with which he is still able to describe episodes of Westphalian country life and academic life is impressive. Already in 1857, in his 2nd semester, he decides to become a university teacher (p. 67). At first, he was enrolled for mathematics and physics. However, he was no longer sure whether his “mathematical disposition was sufficient to be allowed to successfully enter the path of a university lectureship in this subject.” In a chemistry lecture, he met a medical student, Josef Koch from Hildesheim. “We took a liking to each other; he unintentionally became the external reason for me to turn to the study of medicine, especially anatomy.” Both were particularly impressed by Henle's lectures. It is astonishing how Waldeyer switched from mathematics to medicine rather by chance.

In the following 100 pages he describes his years as a university lecturer: Königsberg, Breslau, Strasbourg (*Wanderjahre*), Berlin (*Schlußjahre*). In 1872 he was called to the newly founded University of Strasbourg. Here, as expected, there is a lot of development and pioneering work to be done. “In spite of all these shortcomings and all the effort it took to set up a habitual and orderly structure, I can say that this period was the most precious of my life.” The Berlin period also includes a description of “women's studies and the women's question” (p. 196 ff.). Ultimately, he allows women to study only reluctantly and because of external pressures. “When the question of women's higher education began to arise in Germany, I allowed myself to be persuaded—it was in 1888 at the German Assembly of Physicians and Natural Scientists in Cologne—to give a speech about it, in which I expressed myself on the whole in a negative way. With this—the ladies may forgive the use of the expression which is not meant maliciously—I stirred up a hornet's nest, because in various journals, in anonymous letters and letters with names, I was attacked more or less violently, especially since I also refused women admission to my lectures in anatomy and to the dissection exercises. Later, when women were officially enrolled and given all student rights, I had to admit them.”

His writing on the study of the female is read today as pure anachronism and did not remain without shining contradiction (16). Lina Morgenstern, at that time a famous representative of the German women's movement, countered: “Highly esteemed Professor! If I attempt to write a contradiction to individual points of your presentation concerning the medical study of women, this will occur from the following aspects: The significance of a presentation lies in the topic which is discussed, in the position which the lecturer occupies in the scientific and civilised world, and in the audience which is being spoken to. In all three directions, your presentation is of the utmost importance for the female movement and should not be underestimated. Especially at this time, as there is a mighty energetic movement in our fatherland, from various female circles, which is demanding female doctors for female and paediatric illnesses as a sanitary and moral necessity and therefore aiming for medical study of women in Germany, the lecture from a famous anatomist (…) seems to be like a declaration of war from the enemy camp: even more so, than the doctors who naturally listened to this speech with great approbation, who are the natural opponents of female study” (3, 17). From 1908, the doors to Prussian Universities were open to German women—a cursory and interesting summary of these labour pains and the associated polemics can be found by Arina Völker (18). Women studied medicine from as early as the 1830s in the USA and from 1864 in Switzerland. Prussia, however, would not admit them until 1908, another 9 years after the parliament decision to this effect. Because of this Waldeyer certainly was backward even for his time (2).

Starting on page 200, he then describes the setup of his teaching activities in Berlin with lectures, practical exercises in microscopy and dissection. Here didactic, technical and organisational details are mentioned, which illustrate how much he must have influenced the anatomical teaching at that time and enriched it with new ideas.

In Chapters 7 and 8 (p. 211 ff.), he describes his connections with the academies of science, his membership in scientific, charitable, and social associations, as well as his extracurricular activities and congress trips. Already in 1884, shortly after his appointment to Berlin, he was elected a full member of the Prussian Academy of Sciences. He describes its history (founded in 1700), organisation, and “mode of operation” (p. 211-223). The fact that he became permanent secretary of the Academy in 1896, a post he held until 1919, almost until he reached the age of 83 is a testimony to his reputation, and today one would probably also say to his good networking.

He cites the networking of scholars all over the world, which one of the founding fathers of the Prussian Academy, Leibniz, already had in mind, as one of the main tasks of the academies. Globalisation in the best sense!

At the end of the 19th century, the academies of Vienna, Paris, London, Berlin and others consistently promoted an association of the scientific academies all over the world, certainly also due to improved external circumstances such as optimised means of transportation and better communication possibilities (*Funkspruch und Flugzeuge*, p. 223). This association was officially founded in Wiesbaden in 1899, from the Prussian side under the leadership of Mommsen and Virchow. The general assemblies met at 3-year intervals in Paris (1901), London (1904), Vienna (1907), Rome (1910), and St Petersburg (1913). Berlin had been scheduled for 1916. Due to the intensification of nationalistic tendencies and the turmoil of war, this did not happen (after WW1, it was re-established in 1919 as the International Research Council). Waldeyer remarks that for him scientifically the meeting in London was of special interest (p. 229). At the request of Wilhelm His (Leipzig), with whom he also had a good personal rapport (p. 286 ff.), the Brain Commission was founded. Waldeyer chaired the relevant negotiations. Until his death in 1904, His was its president; he was succeeded by Waldeyer and a number of institutes, called “Interacademic Brain Research Institutes,” were successfully united for work with a common goal [Cajal/Madrid, Flechsig/Leipzig, Edinger/Frankfurt am Main, Obersteiner/Vienna, Monakow/Zurich, Donaldson/Philadelphia, Bechterew/St. Petersburg, Kappers/Amsterdam, and Schaffer/Budapest, p. 231 (19)].

In a kind of conversational tone, Waldeyer skillfully reports in these chapters in a very entertaining manner, enriched with abundant anecdotal information about his academic travels, memberships and many encounters.

Chapter 9 is dedicated to “some colleagues from Berlin and Leipzig.” Robert Koch and Wilhelm His, who he portrays in an affectionate and respectful manner, are particularly noteworthy. Virchow, with whom he obviously had a close and friendly relationship, is already portrayed in several places in Chapter 8. The account of his funeral is impressive (p. 251 ff.). At the end of Chapter 9, he lists the collaborators at his various places of work and an impressive list of international guest professors at the Berlin Institute.

Chapter 10 is devoted to his (mostly) private travels and hikes. Nevertheless, this chapter is also linked to many interesting professional-academic encounters all over the world.

Chapter 11 deals with the “Relations with the Prussian Royal House.” In particular, a lot of attention is paid to the description of the suffering of the Crown Prince and later Emperor Friedrich III (cancer of the larynx). Here, Waldeyer gives his view of things and puts his involvement on record (pp. 322-334). Friedrich was treated for his laryngeal disease by London's Sir Morell Mackenzie, one of the leading laryngologists of his time. According to the German physicians involved, his diagnosis of a benign tumour delayed the (presumably curative) treatment of the disease, to which he succumbed in 1888.

Chapter 12 describes “Political and Wartime Events.” His observation of the historical events is enriched with personal experiences in his private and professional environment, in particular his experiences in military hospitals in the 1870-1871 War. His report ranges from 1848, with his perception of the revolution over Central Europe as a 12-year-old boy, to the Balkan Wars 1912-1913 with the intermediate Crimean War, the Austro-French War and the struggle over the German consensus with the German-French War 1870-1871. Further wars are referred to at the end of the chapter (e.g., Peru-Chile, United States-Spain, Russo-Turkish War, Boxer Rising, Russo-Japanese War). This chapter contains various subjectively influenced opinions. It is therefore not straightforward and must be understood and recognised particularly in context of the historical events, similar to Chapter 13, which covers the World War and the Revolution. Chapter 14 portrays “the picture of Germany before and after the World War.” Dilapidation, destruction and damage, strikes, deterioration of the transport sector, inflation, public and political insecurity are keywords of his gloomy picture of Germany after the First World War. Nevertheless, even here he does not deviate from his trust in a better future, one could almost say in his typical outlook toward life. He again dares to look back at European history, hoping for peace and political stability, which in his opinion could be reached with the “United States of Germany.”

Due to the short amount of time that had passed since the First World War, some of his partially nationalist views which influenced this chapter must therefore be evaluated against the historical background. It is, however, apparent that he is dedicated to providing a differentiated and differentiating representation of events in these “political” chapters (12-14). Naturally, he is subject to errors. On the other hand, he demonstrates a farsightedness and, from a vantage point of the present, an astounding ability for acquisition of political and historical knowledge.

The title of Chapter 15 already suggests that a kind of quintessence of the entire book, possibly even his life, is stipulated here: “Progress in science and technology and its utilisation for our lifestyle since my birth. Conclusion”. The end of this chapter emphasises that his final chapter is meant to raise hope and courage; he is hoping for scientific and global economic advancement. He consciously compares the positive reflections on the substantial progress in his lifetime to the “hardness and distress of the penultimate chapter.” The year of publication of his memoirs (1920)—a few years after the first and on the pre-eve of the Second World War—emphasises the special aura of the last chapter. He could not have anticipated that the catastrophe of the First World War was to be followed by an even greater one and his hopes would only gradually become reality in the second half of the century.

The recital of the progress throughout his 85 years of life (1836-1921) is tremendous and comprises abundant highlights, some of which shall be portrayed here. He commences with biology and medicine: the fact of common components of life, the organic cell, was only determined shortly after his birth (Schleiden and Schwann, Cell Theory 1838, Virchow: omnis cellula e cellula 1855).

Furthermore, he specifies:

- The history of evolution—the study of inheritance—nuclear division
- Anatomy of the nervous system: fine structure, components, neural pathways
- Comparative anatomy
- Discovery of the function of the endocrine glands
- Physiology of the sensory organs
- Physiological chemistry
- Mirror examinations (eye, nose, larynx)
- Technical procedures of the topographical anatomy (Corrosion and frozen section procedure, etc.)
- Microscopic technology and improvement of microscopy
- Anthropology
- Bacteriology
- Antisepsis and asepsis
- Anaesthesia
- In physics: theory of atoms, electrons and ions, electromagnetism, spectral analysis, x-rays, quantum theory, theory of relativity
- In chemistry: fabrication of dye, chemistry of the protein /albumin, explosives, discovery of various new elements (inter alia Radium)
- Miscellaneous in natural sciences and social sciences, technology and agriculture: discovery of new planets, periodic oscillation of the axis of the earth, North and South Pole expeditions, photography, cinematography, phonography, telephone, seismograph, artificial fertilisers, sowing, mowing, threshing machines, steam ploughs, submarines, flight/onset of aviation, typewriter, sewing machine, fire brigade, department stores.

Waldeyer dedicates a longer section—a quasi-exemplary presentation—to the lighting and transportation sector, by describing these in great detail from his youth to his old age. He closes this chapter with the words:

“I will not see this happier future in which our dear fatherland will stand in honour and peace amongst the people on earth, but in firm trust that it shall come, take leave from this life, which has given me so many dear, good and pleasant experiences and close my reminiscences with the wishes that all, who will read them, shall see the happy days of which I can merely dream at this point.

My dawn
Offered niceties in abundance
My day was bright,
A source of life.
On nighttime's mats
Dark shadows fell.
Now softly arrives
The silent night. –
I don't shy away:
Through night to the light!”

## Ovary and Egg

“Ovary and Egg—a contribution to the anatomy and evolution of the sexual organs” was first published in 1870 (20). At this time, Waldeyer was 34 years old and had graduated 8 years previously. It is said to be his most important work and in 2021 reached 18 editions within 150 years. The book contains six picture plates, which are explained in great detail. It is divided into two main parts: anatomy and evolution. In the anatomy part, the ovaries of mammals, birds, reptiles, amphibians and fish are described, after that a chapter “Comparative interpretation of the vertebrate eggs,” followed by a chapter “Ovaries of invertebrates” and an appendix (“Corpus Luteum”). The chapter on mammals initially describes the general findings (pp. 3-18). A separate part with the human ovary (pp.19-30), the ovaries of dogs, cats, rabbits, pigs, cattle and sheep follows (approximately two pages each). The third part of the “mammal chapter” discusses Graaf's follicles and the mammals' egg. The subject of evolution starts with a historical outline, referring to works by Wolff (1759), Oken (1806), Meckel (1808-1812), Müller (from 1829), Jacobsen (1830) and Remak (1854). A detailed view of the Wolffian duct (section 2), Wolffian corpus (section 3), conduct of the germinal epithelium to the peritoneum (section 4), confluences of Wolffian ducts and Müller ducts into the cloaca (section 5), development of the reproductive glands (section 6) and finally the development of the urogenital system of humans and mammals (section 7). The most important scientific finding of this work is summarised in section 8 (“Final Chapter”) and describes how the reproductive system in vertebrates is initially developed as bisexual and is not formed by a mutual neutral asset: “But another, not unimportant point for teratology follows from the observations with the assurance that the genetic assets of each individual are hermaphroditic, even with the highest vertebrates. […] that this opinion is correct, follows from the communicated facts. Every individual has at the same time germinal epithelium as well as the Wolffian duct with its epithelium. But this theory goes further. Those individuals who later become female also have the beginnings of the seminal vesicles in the epoophoron, just as the initial formation of eggs can also be found in the epithelial layer of the subsequent testicle of those individuals who later become male” (p. 152 ff.). This basic finding is culturally important and part of the civilising store of knowledge. It is also the reason that this book is still being printed to this day.

## The Pelvis

Waldeyer's monography “The Pelvis—topographic-anatomical with particular focus on surgery and gynaecology” (21), dated 1898, is a monumental work in several respects. It comprises nearly 700 pages and has been re-printed multiple times, making it available in bookstores, even today. The book expands on the chapter “Pelvis” which Waldeyer contributed to J G Joessel's textbook of topographic-surgical anatomy. Waldeyer completely reworked this chapter and published it as a separate work. The book starts off with definitions and the description of the external appearance followed by a comprehensive description of the bony pelvis (pp. 16-134) as well as of the soft structures of the pelvic wall (pp. 134-146). The chapters “Male pelvis walls according to the individual regions” (Regio sacralis, glutea, inguinalis, perinealis, pubica et pudendalis, ca 80 pages), “Internal topography of the male pelvis” (24 pages) and “male pelvic viscera” (158 pages) follow. These are followed by the corresponding chapters of the female anatomy (18 pages, 3 pages, ca 180 pages), continuing with two subchapters (“Fasciae pelvis” and “Herniae perineales et endopelvinae,” 20 pages). The book concludes with three additional chapters (“Development of the pelvic viscera,” “Malformations,” and “Surgical Anatomy,” 35 pages). The index of keywords comprises 16 pages; the book contains ~120 images and a comprehensive bibliography. Neither before nor since then has a more comprehensive work been composed regarding this section of the body. Although many parts may now be outdated, significant topographic-anatomical parts are still fundamental. Waldeyer attempted to summarise all existing knowledge in his work and also added his own essential discoveries with partially new terms. Apart from that his work follows the latest nomenclature of the time (Basler Nomina Anatomica, BNA) in 1895. In accordance with contemporary thinking, Waldeyer attempted to determine “racial differences” by the pelvic viscera (e.g., p. 12, 99), in this work too, which seems rather disconcerting today.

Due to the many images, numerous structured summaries, charts and graphs a very comprehensive and detailed view of this region of the body was produced. In many parts he includes changes related to physiology, functionality, pathology and aspects of ageing. Additionally, he also tries to link the clinical disciplines, not only by emphasising the surgical anatomy, which is clearly apparent in his description of the pelvic fractures (incl. specific nerve damage), but also the various types of hernias, tumours, the gestational anatomy, the depictions of clinical-diagnostic and investigatory aspects and the involvement of imaging techniques (e.g., *Kystophotographischer Atlas* by M. Nitze). This considerable compression of the subject has resulted in the work, in particular the topographic part, still being of great interest even today.

## Discussion and Conclusion

Waldeyer was one of the most prominent anatomists and scientists of his time. Due to his numerous and partly still valid contributions to date, Waldeyer can be considered a pioneer. His scientific interests were widely strewn and he can justifiably be described as a generalist. His scientific merits are not only restricted to his original research contributions but, in fact, also include his ability to succinctly summarise scientific concepts and constructs and publish these.

But even Waldeyer was subject to mistakes, succumbed, sometimes uncritically, to the trends of his time, wrongly evaluated political and historical developments and was overtaken by history concerning some of his views (race theory, question of women's rights). On the other hand, he was clearer sighted and, due to his extensive education and high intelligence, sensitive and differentiating. Already during his lifetime his teaching performance prepended his research performance significantly. Even his memoirs reflect that he was an innovative and committed teacher of anatomy, who also enhanced the educational concept of anatomy with many productive ideas. But it would be wrong to diminish his performance as a researcher.

At many points in his memoirs, his language develops a radiance and his descriptions become very intense, making his work worth reading even today. More or less throughout the entire work, there are signs of his talent as a prose author. Multiple insights into the scientific and institutional networking and the increasing development of scientific (professional) societies become apparent. His heritage will remain as the person to coin the terms “neuron” and “chromosome,” in the evolution of the neuron theory and in his “large” monographies.

As a comparatively young man he presented “Ovary and Egg” in 1870. This work provides a summary of the existing knowledge of gametes and multiple additional contemporary finding together with comparative anatomy and evolution. His real and main significance lies in the developmental biological discoveries of the concept of the constitutional bisexuality, which many scientists categorise as a world intellectual heritage.

His monography “The Pelvis” dated 1898 impresses with its complex content and large scope. To the present day, it has remained a fundamental work for various aspects of topographic and functional anatomy. It is considered to be one of the most complete works in the field of topographic anatomy. It is truly a rich source of interesting facts for both specialist anatomists and practical physicians (1). These highlights from the medical-historical literature are still informative today and present Waldeyer as an interesting illustrious physician and research personality.

## Author Contributions

HS designed the study, analysed the literature, and wrote the main part of the manuscript. CP-K and FK analysed the literature, wrote parts of the manuscript, and revised the manuscript. All authors contributed to the article and approved the submitted version.

## Conflict of Interest

The authors declare that the research was conducted in the absence of any commercial or financial relationships that could be construed as a potential conflict of interest.

## Publisher's Note

All claims expressed in this article are solely those of the authors and do not necessarily represent those of their affiliated organizations, or those of the publisher, the editors and the reviewers. Any product that may be evaluated in this article, or claim that may be made by its manufacturer, is not guaranteed or endorsed by the publisher.
